# *Jnk2* Effects on Tumor Development, Genetic Instability and Replicative Stress in an Oncogene-Driven Mouse Mammary Tumor Model

**DOI:** 10.1371/journal.pone.0010443

**Published:** 2010-05-03

**Authors:** Peila Chen, Jamye F. O'Neal, Nancy D. Ebelt, Michael A. Cantrell, Shreya Mitra, Azadeh Nasrazadani, Tracy L. Vandenbroek, Lynn E. Heasley, Carla L. Van Den Berg

**Affiliations:** 1 Division of Pharmacology and Toxicology, Center for Molecular and Cellular Toxicology, College of Pharmacy, The University of Texas at Austin, Austin, Texas, United States of America; 2 Department of Craniofacial Biology, University of Colorado Denver Anshutz Medical Campus, Aurora, Colorado, United States of America; 3 Institute of Cellular and Molecular Biology, School of Biological Sciences, The University of Texas at Austin, Austin, Texas, United States of America; Roswell Park Cancer Institute, United States of America

## Abstract

Oncogenes induce cell proliferation leading to replicative stress, DNA damage and genomic instability. A wide variety of cellular stresses activate c-Jun N-terminal kinase (JNK) proteins, but few studies have directly addressed the roles of JNK isoforms in tumor development. Herein, we show that *jnk2* knockout mice expressing the Polyoma Middle T Antigen transgene developed mammary tumors earlier and experienced higher tumor multiplicity compared to *jnk2* wildtype mice. Lack of *jnk2* expression was associated with higher tumor aneuploidy and reduced DNA damage response, as marked by fewer pH2AX and 53BP1 nuclear foci. Comparative genomic hybridization further confirmed increased genomic instability in PyV MT/*jnk2−/−* tumors. *In vitro*, PyV MT/*jnk2−/−* cells underwent replicative stress and cell death as evidenced by lower BrdU incorporation, and sustained chromatin licensing and DNA replication factor 1 (CDT1) and p21^Waf1^ protein expression, and phosphorylation of Chk1 after serum stimulation, but this response was not associated with phosphorylation of p53 Ser15. Adenoviral overexpression of CDT1 led to similar differences between *jnk2* wildtype and knockout cells. In normal mammary cells undergoing UV induced single stranded DNA breaks, JNK2 localized to RPA (Replication Protein A) coated strands indicating that JNK2 responds early to single stranded DNA damage and is critical for subsequent recruitment of DNA repair proteins. Together, these data support that JNK2 prevents replicative stress by coordinating cell cycle progression and DNA damage repair mechanisms.

## Introduction

Numerous oncogenes including ErbB2, EGFR (Epidermal Growth Factor Receptor), and IGF-1R (Insulin-like Growth Factor 1 Receptor) have been therapeutically targeted in the past two decades. The tyrosine kinase receptors (TKRs) increase activity of the PI3K (Phosphatidylinositol 3-Kinase) and Ras/Shc/MAPK pathways to enhance cell proliferation and suppress apoptosis. Ironically however, oncogene induced replicative or oxidative stress can also result in senescence as a barrier to tumor progression [1]. Understanding the differential signaling mechanisms that influence proliferative versus senescent outcomes is essential for inhibiting oncogene driven tumor growth without adversely affecting tumor barriers. JNK is frequently a downstream mediator of TKR responses. The Polyoma Middle T Antigen (PyV MT) oncogene activates PI3K and Shc/MAPK dependent pathways, and induces c-Jun phosphorylation (via JNK activation) and transcriptional activity [2]. Moreover, the Met oncogene mediates transformation via PI3K and JNK [3]. These studies maintain that TKR induced JNK/c-Jun activity enhances tumorigenesis. C-Jun may be essential to this process since *c-jun* deficient cells undergo premature senescence resulting from DNA damage accumulation and inefficient repair [4]. Targeting c-Jun for cancer prevention or treatment presents many challenges given its ubiquitous function in cells. In contrast, the upstream mediators of c-Jun may be attractive targets. To date, no studies have addressed the specific functions of the various JNK proteins in TKR mediated cancer progression. The PyV MT mouse mammary tumor model closely emulates both early and late stages of human breast cancer and serves as an excellent model to address such questions [5].

JNK proteins are known mediators of growth factor responses but this area is understudied compared to other types of stimuli. Notably, JNKs convey downstream messages from a wide-variety of important cancer related proteins including Ras, PI3K, Rac1, and PTEN (Phosphatase and Tensin homolog) [6], [7]. JNK proteins were thought to be required for Ras mediated transformation *in vitro* but were found unnecessary in an *in vivo* model using Ras transformed compound *jnk1−/−/jnk2−/−* 3T3 fibroblasts [8]. Inhibition of basal JNK activity in established breast cancer cell lines leads to cell cycle aberrations and endoreduplication [9]. These data support the need to mechanistically study the involvement of JNK proteins using spontaneous tumor models.

While JNK proteins are generally considered stress induced kinases, understanding the biological contributions of the three *jnk* genes and resulting ten isoforms has been challenging. In many instances genetic knockout and shRNA approaches are needed to elucidate the specific functions of the products of the three *jnk* genes. While compound knockout of *jnk1* and *jnk2* is embryonic lethal, single knockouts are viable, suggesting that *jnk1* and *jnk2* may possess redundant functions during development. MEFs (Mouse Embryo Fibroblasts) are largely used to study the specific and combined *jnk1* and *jnk2* mediated phenotypes and signaling pathways. These studies have formed the basis of our knowledge for the diverse roles of JNK proteins. However, tissue specific models are required to recapitulate pathogenesis of various diseases including cancer, metabolic, cardiovascular and neurological diseases. Moreover, animal models are essential in providing information on processes such as susceptibility to tumorigenesis. Studies using single *jnk1* or *jnk2* knockout mice have provided insight into isoform specific functions of JNK proteins in diseases like leukemia, skin tumorigenesis and insulin resistant diabetes [10], [11], [12], [13], [14]. In these cases, disparate phenotypes between *jnk1* and *jnk2* knockouts were observed.

JNK proteins phosphorylate diverse substrates but many of JNKs' functions are thought to revolve around their ability to phosphorylate c-Jun and induce AP-1 dependent transcription. When phosphorylated, c-Jun participates in the AP-1 dimer, increasing gene expression of c-myc and cyclin D in mammary epithelial cells [15]. Overexpression of non-phosphorylatable c-Jun has demonstrated important functions in both cancer and mammary gland development due to proliferation defects [15]. By phosphorylating substrates other than c-Jun, including IRS-1, Bcl-2 related proteins or FOXO transcription factors, JNK proteins play integral roles in a variety of cellular responses like cell death and senescence [16], [17].

Here, we show that systemic deletion of *jnk2* in the PyV MT transgenic mouse model shortens tumor latency and increases tumor multiplicity. Additionally, PyV MT*/jnk2−/−* mammary tumors have a significantly higher frequency of aneuploidy than the PyV MT*/jnk2+/+* controls but show reduced DNA damage response markers. *In vitro* studies comparing PyV MT/*jnk2−/−* and PyV MT/*jnk2+/+* cells show that re-initiation of the cell cycle is associated with increased p21^Waf1^ expression and cell death, with minimal changes in p53 response. Overexpression of CDT1 further confirms that PyV MT/*jnk2−/−* are more susceptible to replicative stress and subsequent cell death. In summary, our data unveil important functions for *jnk2* in tumorigenesis, replicative stress response and cancer cell survival.

## Results

### *Jnk2* knockout shortens tumor latency and increases tumor multiplicity induced by the PyV MT transgene

**Figure 1:**
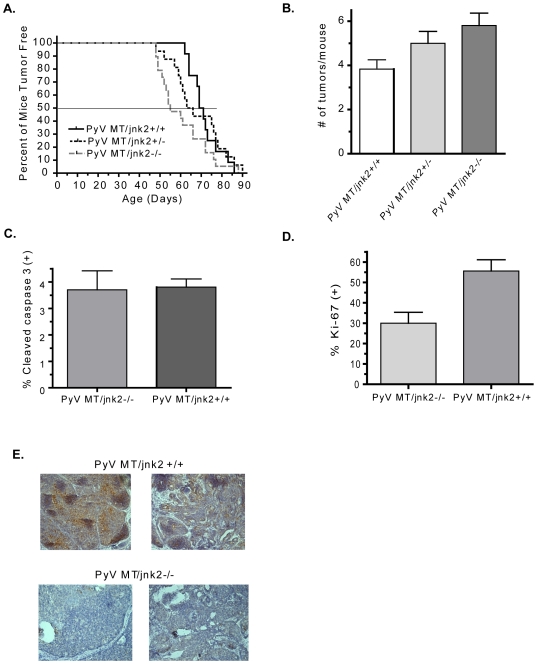
Systemic *jnk2* deletion enhances tumor development. PyV MT*/jnk2+/+* (n = 12), PyV MT/*jnk2+/−* (n = 16), and PyV MT/*jnk2−/−* (n = 19) mice were palpated for mammary tumors thrice weekly. Once palpated, tumor growth was recorded thrice weekly. **A**). Kaplan Meier graph showing age of first tumor palpation (median age was day 55 for PyV MT*/jnk2−/−* vs. day 70 for PyV MT*/jnk2+/+*, p = .11); **B**). Total number of tumors palpated per mouse at the time of harvest was higher in PyV MT/*jnk2−/−* mice compared to PyV MT/*jnk2+/+* mice, p = 0.0192); **C**). Paraffin embedded, non-target tumor sections were probed with cleaved caspase 3 primary antibody and detected using FITC labeled secondary antibody. Nuclei were stained with propidium iodide. The total number of cells staining positive for cleaved caspase 3 were scored and divided by the total number of nuclei (n = 5 tumors in each group); **D**). Paraffin embedded tissue sections were probed with Ki-67 primary antibody and detected using DAB. Cells staining positive for Ki-67 were counted and divided by the total number of nuclei (Hematoxylin) per field. Five fields per tumor were counted (n = 5 per genotype, p = 0.0159); **E**). Paraffin embedded tissue sections were probed with p-c-Jun (Ser63) primary antibody and detected using DAB. Hematoxylin was used as a nuclear stain.

In the studies presented herein, we set out to assess the contributions of JNK2 isoforms in mammary tumorigenesis and metastasis using the MMTV-PyV MT transgenic mouse model [18]. PyV MT mice were backcrossed to the Balb/C strain for over 10 generations and were then mated with *jnk2−/−* mice to obtain PyV MT*/jnk2+/+,* PyV MT*/jnk2+/−* and PyV MT/*jnk2−/−* genotypes. Female transgenic mice were palpated for tumors three times weekly. During the time of observation, PyV MT/*jnk2−/−* mice developed palpable tumors earlier than the PyV MT/*jnk2+/+* mice (median time to first tumor palpation, T_50_ = day 55 vs. day 70, respectively). PyV MT/*jnk2+/−* mice experienced an intermediate latency, demonstrating that tumor latency increased incrementally with *jnk2* expression (Figure 1A). Importantly, PyV MT*/jnk2−/−* mice also experienced significantly higher numbers of tumors per mouse (i.e. tumor multiplicity), and the heterozygous mice showed an intermediate tumor multiplicity (Figure 1B). These data support that loss of *jnk2* expression facilitates tumorigenesis by shortening tumor latency and increasing tumor multiplicity.

Assessment of tumor apoptotic indices using cleaved caspase 3 immunohistochemistry showed no difference between the PyV MT/*jnk2+/+* and the PyV MT/*jnk2−/−* tumors (Figure 1C). In contrast, the percent of cells staining positive for Ki-67, a marker of cell proliferation, was significantly higher in the PyV MT/*jnk2+/+* tumors compared to the PyV MT/*jnk2−/−* (Figure 1D). This finding correlated with the intensity and frequency of phosphorylated c-Jun in tumor cells which was notably higher in the PyV MT*/jnk2+/+* tumors (Figure 1E). Together, these data support that the loss of *jnk2* expression facilitates tumorigenesis as shown by shortened latencies and higher tumor multiplicity. However, once tumors developed the *jnk2* knockout tumors showed less cell proliferation and reduced c-Jun phosphorylation.

### Absence of *jnk2* increases tumor aneuploidy

**Figure 2:**
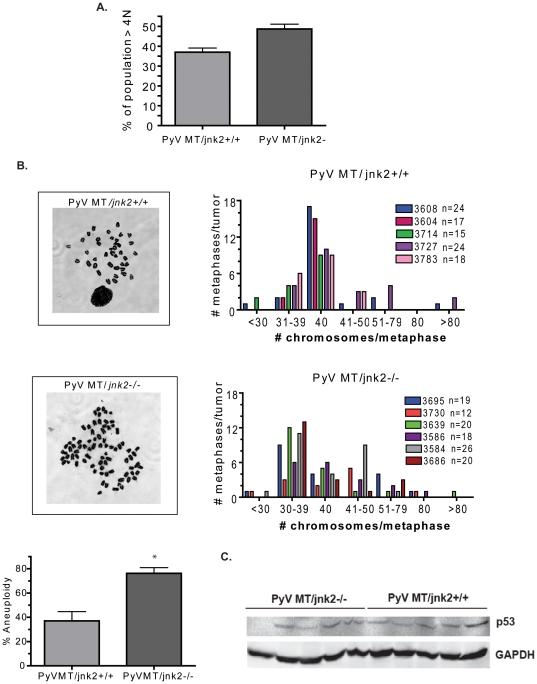
Target tumors obtained from PyV MT/*jnk2*−/− had increased aneuploidy. **A**). Tumors were finely minced and digested as described in the Methods section. Cells were trypsinized at passage 2 to 3 and assessed for DNA content using PI staining. PyV MT*/jnk2−/−* tumors contained more cells with DNA content ≥4N (PyV MT/*jnk2+/+* (n = 5), PyV MT/*jnk2−/−* (n = 10), p = 0.0485); **B**). Primary cells were treated with colcemid and harvested to assess number of chromosomes per metaphase. The number and frequency of abnormal (aneuploid) chromosome numbers are higher in PyV MT*/jnk2−/−* compared to PyV MT/*jnk2+/+* (p = 0.0043). Each colored bar represents a single target tumor obtained for an individual mouse. The number to the right of the mouse number denotes the number of metaphases counted for each target tumor. The graph illustrates the frequency of a particular chromosome number for each target tumor; **C**). Tumor lysates were subjected to SDS PAGE and western blotting using p53 primary antibody and detected using chemiluminescence. GAPDH primary antibody was used to assess similar loading amongst samples.

We then focused our studies more closely on the potential mechanism(s) by which *jnk2* deletion enhances tumorigenesis. Loss of cell cycle checkpoints during replication can result in amplification or deletion of various genes and genomic instability. Moreover, inhibition of basal JNK causes endoreduplication in breast cancer cell lines [9]. Given that tumor development was facilitated in PyV MT/*jnk2* knockout mice, we evaluated whether there was a difference in ploidy between the PyV MT*/jnk2+/+* and the PyV MT*/jnk2−/−* tumors. To this end, tumors were harvested and primary mammary tumor cells were cultured. Early passage primary tumor cells (passages 2 or 3) were harvested and processed for cell cycle analysis using propidium iodide (PI) staining. PyV MT*/jnk2−/−* tumors showed significantly higher percentages of cells with ≥4N DNA content compared to the PyV MT*/jnk2+/+* tumors (Figure 2A), consistent with the presence of tetraploid or aneuploid tumor cells in the *jnk2* deficient tumors.

Cell cycle analysis using PI staining does not allow discrimination between 4N diploid and 2N tetraploid populations of cells and is also unable to detect losses or gains of only a few chromosomes. Therefore, the number of chromosomes in each metaphase spread was counted using the same set of tumors. Figure 2B illustrates that the number of chromosomes per metaphase in the PyV MT*/jnk2+/+* tumors was more frequently diploid compared to the PyV MT*/jnk2−/−* tumors. Each tumor is represented by a specific color (listed as mouse number and number of metaphase spreads counted per tumor in the legend). While aneuploidy was rather common in both groups, it was significantly more frequent in the PyV MT*/jnk2−/−* tumors. Together, these data are consistent with the conclusion that loss of *jnk2* expression increases tumor aneuploidy in this model.

Loss of p53 function frequently leads to tumorigenesis, and aneuploidy contributes to this effect. Mutations or deletion of p53 often precede aneuploidy. Mutant p53 protein in more stable and frequently shows notably higher expression than wildtype p53 protein. Thus, p53 expression was measured to determine if its deletion or mutation is associated with the aneuploidy observed in the *jnk2* knockout tumors. Western blot analysis of PyV MT tumor lysates showed that p53 expression was very low and similar with both genotypes (Figure 2C). These data are consistent with wildtype p53 expression and indicate that p53 expression is not changed in the absence of *jnk2*. Therefore, genetic deletion or mutation of p53 is not likely contributing to aneuploidy in this model.

### pH2AX and 53BP1 DNA damage foci are less frequent in PyV MT*/jnk2−/−* tumors

Alternatively, DNA damage due to oncogene driven proliferation can impair cell cycle checkpoints and/or result in oxidative stress. Given the increase in aneuploidy in the PyV MT*/jnk2−/−* tumors, we evaluated these tumors for signs of DNA damage. ATM phosphorylates Ser139 of H2AX in response to double strand breaks (DSB). Phosphorylated H2AX (pH2AX) then localizes to DSBs and recruits other proteins involved in DNA damage recognition and repair [19], [20]. Checkpoint recovery occurs when cells re-initiate cell cycle progression. This latter response is mediated by various phosphatases which act upon proteins including pH2AX, see ref [19] for review. JNKs were recently shown to directly phosphorylate H2AX on Ser139 [21]. Therefore, we hypothesized that pH2AX would be reduced in *jnk2* knockout tumors.

**Figure 3:**
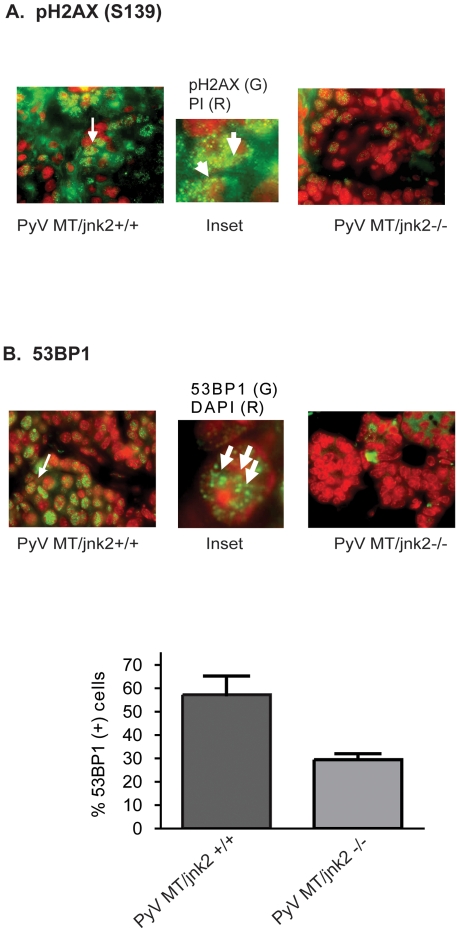
pH2AX and 53BP1 staining in PyV MT tumors. **A**). Paraffin embedded PyV MT tumors were stained with pH2AX antibody (green). Nuclei were counter stained with propidium iodide (PI, red); Inset shows high magnification of a cell from a PyV MT/*jnk2+/+* tumor with arrows pointing at foci. **B**). Tumor sections were processed as in A) except incubated with 53BP1 primary antibody. Images were captured and pseudo-colored. 53BP1 antibody (red) and DAPI nuclear stain (green). Inset shows high magnification of a cell from the PyV MT/*jnk2+/+* with arrows pointing at foci. **C**). Cells with ≥4 nuclear 53BP1 foci were counted as positive cells in each field; a minimum of 5 fields were counted/mouse. Each point represents a tumor average (p = 0.0286).

Paraffin embedded tumors were probed with a pH2AX primary antibody. PyV MT/*jnk2+/+* tumors showed a notably higher frequency of H2AX foci (individual foci could not be resolved). In comparison, pH2AX foci in the PyV MT*/jnk2−/−* tumors were much less frequent (Figure 3A). These data may be interpreted in three ways, 1). DNA damage is less common in the PyV MT*/jnk2−/−* tumors; 2). the PyV MT*/jnk2−/−* tumors have fewer foci due to loss of JNK2 phosphorylation of pH2AX; or 3). the PyV MT*/jnk2−/−* tumors show less DNA damage response and/or a more rapid checkpoint recovery even in the presence of clear differences in aneuploidy.

53BP1 is a key component in DNA damage checkpoint and functions as a barrier during early tumorigenesis. 53BP1 localizes to DNA lesions initially recognized by pH2AX but inactivation of 53BP1 and p53 occurs with tumor progression and decreased apoptosis [22]. To validate our pH2AX findings and to further address the presence of DNA damage or DNA damage response, we probed tumor sections with a primary 53BP1 antibody and counted the fraction of cells with multiple 53BP1 nuclear foci (Figure 3B). Again, the PyV MT*/jnk2+/+* tumors demonstrated a significantly higher fraction of cells with 53BP1 foci (Figure 3C). These data support that PyV MT*/jnk2+/+* tumors show either increased DNA damage or increased repair response to DNA damage and cell cycle resumption compared to the PyV MT*/jnk2−/−* tumors. These data are also consistent with the reduced proliferation observed in *jnk2* knockout tumors shown in Figure 1.

### Genetic deletions and amplifications occur more frequently in PyV MT/*jnk2−/−* tumors

Next, we set out to further validate whether genomic instability was higher in the PyV MT*/jnk2−/−* tumors. During tumorigenesis, error prone replication leads to DSBs and allelic imbalances [22]. Given that JNK and ATM phosphorylate H2AX, and that pH2AX commonly initiates a DNA damage response to allow repair, we questioned if the PyV MT*/jnk2−/−* tumors harbor more genetic mutations as a result of impaired pH2AX induced DNA damage response or repair. Genomic DNA was isolated from PyV MT*/jnk2+/+,* PyV MT*/jnk2−/−* tumors and nontumorigenic *jnk2−/−* and wild type mammary glands for comparison of gene copy number changes using aCGH (array Comparative Genomic Hybridization). Both tumor genotypes showed gene deletions and/or amplifications compared to their *jnk2* matched normal mammary glands (Table 1), consistent with the presence of genomic instability we observed in Figure 2. PyV MT*/jnk2−/−* tumors showed additional chromosome segments that were amplified or deleted compared to the PyV MT*/jnk2+/+* tumors. Certain segmental changes were observed in multiple pairs (similar segments are marked by common symbols, Table 1), indicating that specific chromosome segments are targeted due to functional significance (including growth selection) or unknown structural similarities.

**Table 1:** aCGH analysis showing segmental amplifications or deletions.

Chip No.	Green Label	Red Label	Segment	Segment	Segment	Segment	Segment	Segment
8802	PyVMT/*jnk2+/+*	*jnk2+/+* Ctl	**Ch 5** ^†^Del 94,530–96,030k	**Ch 13** ^‡^Del65,550–67,050k	**Ch 14** ^§^Del3,150–5,250k			
4702	*jnk2−/−* Ctl	PyVMT/*jnk2−/*−	**Ch 3**Del 106,290–106,410k	**Ch 4** *Del111,570–113,790k	**Ch 7** ^ψ^Del7,050–7,410k^ψ^Del 13,620–14,820k	**Ch 14** ^Л^Del40,260–43,500k		
0302	PyVMT/*jnk2+/+*	PyVMT/*jnk2−/−*	Ch 4*Amp 121,230–121,650k	**Ch 5** ^†^Del94,470–96,030k	**Ch 13** ^‡^Del65,460–67,500k			
3202	PyVMT/*jnk2+/+*	PyVMT/*jnk2−/−*	**Ch 17** ^Ω^Del 6,210–6,390kDel 12,900–13,380kDel 16,710–17,010k	**Ch X** ^π^Del 24,450–25,950k				
4502	*PyVMT/jnk2+/+*	PyVMT/*jnk2−/−*	**Ch 2**Del 174,660–177,540k	Ch 4*Del 60,180–60,900k*Del111,540–113,460k*Del 121,230–121,650k	**Ch 5**Del 11,250–11,910k Amp 104,820–105,060k	**Ch 14** ^§^Del3,150–5,550k^Л^Del40,350–43,350k	**Ch 17** ^Ω^Amp 6,210–6,390k	**Ch X** ^π^Del23,370–30,350k
7802	PyVMT/*jnk2+/+*	PyVMT/*jnk2−/−*	**Ch 3**Del93,900–94,140k	Ch 4*Amp60,180–61,380k*Amp 112,380–113,820k *Amp 121,350–121,650k	**Ch 5** ^†^Del 94,740–95,700k	**Ch 7** ^ψ^Del 7,020–8,940k^ψ^Del 13,620–14,820k	**Ch 13** ^‡^Del66,060–67,260k	

**Figure 4:**
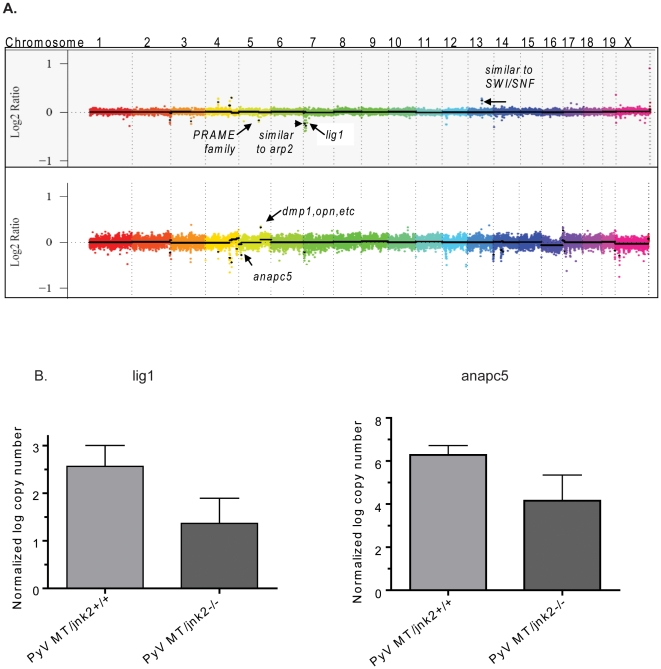
Chromosomal and gene expression changes occur in PyV MT/*jnk2*−/− tumors. **A**). aCGH analysis of PyV MT/jnk2−/− tumors show deletions in segments containing *lig1, anacp5* and amplifications in *dmp-1*, amongst others, compared to PyV MT/*jnk2+/+* controls; **B**). Expression differences between PyV MT/*jnk2+/+* and PyV MT*/jnk2−/−* tumors were compared for *lig1* and *anapc5* using qPCR amplification and standard curves (n = 5 per genotype).

Subsequently, we focused on chromosome segments that were hybridized by five or more probes and consistent with gene copy number changes of one copy number or more. Table 2 shows genes of interest (i.e. genes which have a role in cancer) resulting from this analysis. Namely, DNA Ligase 1 (*lig1*) and Anaphase Promoting Complex 5 (*anapc5*) gene copy numbers were reduced by one copy or more in PyV MT*/jnk2−/−* tumors (Figure 4A). This is consistent with either a complete loss of expression in some but not all cell populations or a one copy number loss throughout most of the tumor cells since samples are heterogeneous. Validation using qPCR confirmed these findings and measured approximately 50% reduction of *lig1* and *anapc5* in PyV MT*/jnk2−/−* tumors (Figure 4B). Similarly, an increase in *dmp-1* expression was validated in the PyV MT*/jnk2−/−* compared to PyV MT*/jnk2+/+* tumors (data not shown). The expression difference in *sod2* (superoxide dismutase 2) was not different using qPCR analysis. aCGH also indicated amplification of a portion of the *nrg3* (neuregulin 3) gene but we were unable to amplify the product using qPCR, presumably due to low abundance.

**Table 2:** Genes of interest on chromosomal segments.

Chromosome	Segment	Genes of Interest
**2**	174.7–177.5 M	Similar to KRAB and zinc finger, C2HS type domain containing protein
**4**	60–61 M	-
	111–114 M	Butyrophilin
	121.3–121.7 M	-
**5**	11–12 M	Anapc5
	94–96 M	PRAME family
	104–105 M	Protein phosphatase, non-receptor type 13Hydroxysteroid (17-beta) dehydrogenase 14Dentin sialophosphoprotein, Dentin matrix protein 1, OPN, MEPESPARC-like 1 (mast9, hevin)
**7**	7–9 M	Similar to actin-related protein 2
	13–14 M	Ligase I, DNA ATP dependent
**13**	65 M–67 M	Similar to SWI/SNF chromatin remodeling protein subfamily e, Similar to High mobility group protein 1 (HMG-1) (Amphoterin)
**14**	3–4 M	-
	40–43 M	Neuregulin 3 (in part)
**17**	6–6.5 M	Protein phosphatase, regulatory (inhibitor) subunit 2
	12.9–13.4	IGF2 receptor,MAS1 oncogene,Wilm's tumor-associated protein,SOD2
**X**	23.4–30.4 M	-

Other genes were identified that show similarity to known gene families which play integral roles in DNA damage response and replication, namely, SWI/SNF, *arp2* (actin related protein 2) and PRAME (PReferentially expressed Antigen of MElanoma) (Table 2). Overall, aCGH analysis confirmed additional gene amplifications or deletions in the PyV MT*/jnk2−/−* tumors compared to normal mammary controls and the PyV MT*/jnk2+/+* tumors. Some of the genetic changes were observed in multiple tumor comparisons. Specifically, *lig1* and the *arp2* related gene were deleted in PyV MT/*jnk2−/−* tumor in comparison to *jnk2−/−* mammary gland and PyV MT*/jnk2+/+* tumors. Also, the SWI/SNF-type gene and the PRAME family of genes differed in three pairs, including the normal mammary comparison. Interestingly, Gorgoulis et al. proposed that certain loci may be prone to DSBs which then lead to genomic instability [22]. Our studies support the proposition that specific loci are vulnerable during certain types of DNA damage. In comparison to normal mammary tissue, DNA damage is higher in PyV MT*/jnk2−/−* tumors.

### Replicative stress is more evident in PyV MT*/jnk2−/−* cells

Cell lines were established from *jnk2* wildtype and knockout tumors to evaluate the role of JNK2 in replicative stress and DNA damage. Subsequent characterization of the cell lines showed that both lines are aneuploid. Genotyping confirmed the presence of the PyV MT transgene, but PyV MT RNA and protein expression were not detected using qPCR or western blot analysis (data not shown), consistent with PyV MT gene silencing. Importantly, cell proliferation did not differ widely between the cell lines when cultured in 10% fetal bovine serum (FBS) containing medium (data not shown).

**Figure 5:**
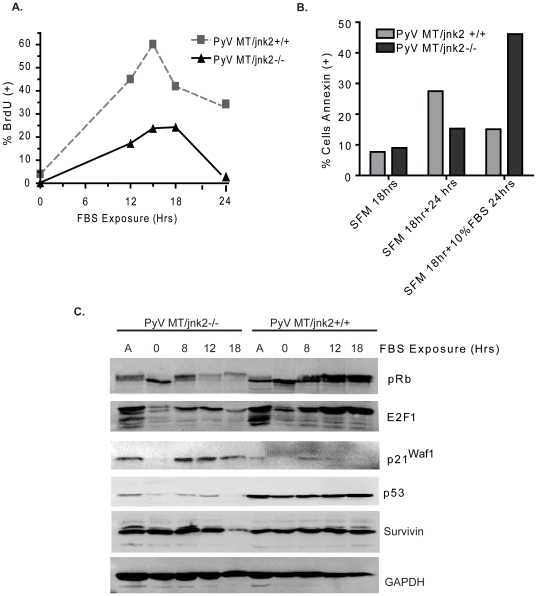
Serum treatment of G_1_ arrested cells induces cell death in PyV MT/*jnk2*−/− cells. **A**). PyVMT/*jnk2+/+* and PyVMT*/jnk2−/−* cells were serum starved for 24 hours and then treated with 10% FBS containing medium. Serum stimulated cells were pulsed with BrdU two hours prior to harvesting and then stained with BrdU primary antibody followed by BrdU detection using flow cytometry. BrdU positivity data are presented as percent positive cells in total cell population; **B**). PyVMT/*jnk2+/+* and PyVMT*/jnk2−/−* cells were serum starved for 24 hours and then treated with 10% FBS containing medium. After 24 hours of serum starvation, cells were either cultured in fresh SFM or medium containing 10% FBS and harvested 24 hours later. Cells were evaluated for Annexin positivity using flow cytometry. Data are expressed as percent positive cells of the total population; **C**). Cells were serum starved as above and then harvested at indicated time points after 10% FBS stimulation to assess expression of various cell cycle associated proteins using western blot analysis with primary antibodies directed towards the indicated proteins. GAPDH was used to compare even sample loading.

To specifically address the role of JNK2 in replicative stress, cells were serum starved for 24 hours and then stimulated with 10% FBS. Cells were pulsed with bromodeoxyuridine (BrdU) for two hours prior to harvesting. DNA BrdU incorporation was then measured using flow cytometry. PyV MT*/jnk2+/+* cells showed approximately three-fold higher BrdU uptake for 12–18 hours after addition of FBS (Figure 5A) which then decreased at 18–24 hours, consistent with transit into G2/M. In contrast, PyV MT*/jnk2−/−* cells showed lower BrdU incorporation which then became negligible after 24 hours, showing that a smaller percentage of cell successfully transited through S phase. Interestingly, the PyV MT*/jnk2−/−* morphologically appeared to undergo cell death 18–24 hours after serum addition. Indeed, FBS treated PyV MT*/jnk2−/−* cells experienced higher Annexin positive staining compared to the controls, untreated PyV MT*/jnk2−/−* cells and the FBS treated PyV MT/*jnk2+/+* cells (Figure 5B).

In light of these observations, the cells were treated in the same fashion and harvested at several time points and compared to asynchronously growing cells to evaluate expression of various cell cycle associated proteins. Both cell lines showed phosphorylation shifts of Rb protein (pRB) and increased expression of E2F1 associated with G1 to S phase transit in response to FBS treatment but the expression of pRb and E2F1 (and other E2F proteins) was lower in the PyV MT*/jnk2−/−* cells (Figure 5C). Notably, PyV MT*/jnk2−/−* cells also showed higher and more sustained p21Waf1 expression than the PyV MT*/jnk2+/+* cells after FBS treatment; whereas p53 expression was higher in PyV MT*/jnk2+/+* cells. These data indicate that absence of *jnk2* prevents cell cycle re-initiation and/or S-phase transit. Increased p21Waf1 expression is consistent with cell cycle slowing or arrest, however p53 does not show the predicted increase in abundance necessary to induce DNA repair and enhancement of p21Waf1 expression in PyV MT*/jnk2−/−* cells.

**Figure 6:**
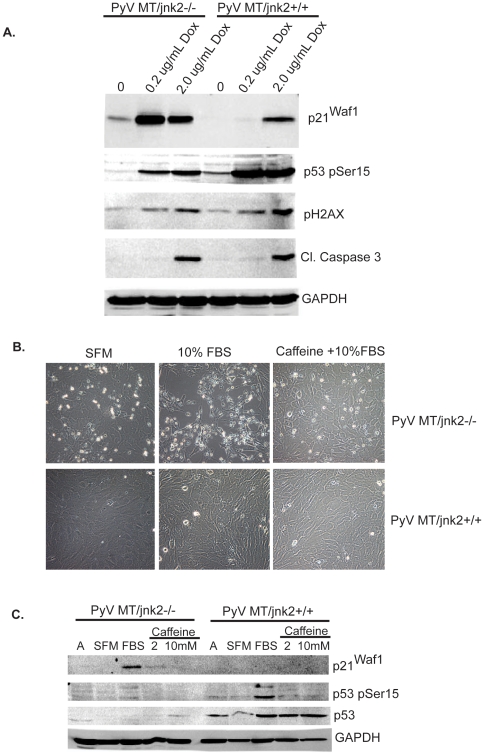
PyV MT/*jnk2*−/− cellular response is specific to replication and reversed by ATM/ATR inhibitor caffeine. **A**). PyVMT/*jnk2+/+* and PyVMT/*jnk2−/−* cells were treated with doxorubicin at the indicated concentrations for 18 hours and then lysed. Expression of p21^Waf1^, p-p53 (Ser15), pH2AX (Ser139), and cleaved caspase 3 were evaluated using western blot analysis. GAPDH was used to compare even loading amongst samples; **B**). Cells were treated as described in A). except caffeine 2 mM was added as indicated; **C**). Cells were treated as described in A). except caffeine 2 and 10 mM were added as indicated. Expression of p21^Waf1^, p-p53 (Ser 15), and p53 were evaluated using western blot analysis. GAPDH was used to compare even loading amongst samples.

The higher expression of p53 in PyV MT*/jnk2+/+* cells may be consistent with lack of p53 response in PyV MT/jnk2−/− cells or expression of mutant p53 in the jnk2 wildtype cells. To functionally evaluate p53, cells were treated with the DNA damaging agent doxorubicin (which induces DSBs and ATM response) for 18 hours [*23*]. Figure 6A shows that both cell lines show enhanced phosphorylation of p53 Ser15, the specific residue phosphorylated by ATM/ATR (albeit higher in the PyV MT*/jnk2+/+* cells). Phosphorylation of H2AX was similarly observed in both cell lines, indicating that ATM or ATR is likely mediating this effect even in the *jnk2*−/− cells. Expression of p21Waf1, a p53 transcriptional target, was also induced by doxorubicin treatment, but the induction of expression was higher in *jnk2*−/− cells especially relative p53 phosphorylation. Despite these differences, both cell lines showed similar apoptotic responses to doxorubicin as indicated by cleavage of caspase 3. These data support that both cell lines express functional p53 and phosphorylation of ATM/ATR substrates such as p53 and H2AX, in response to DNA damage. Again, the PyV MT/*jnk2*−/− cells showed more robust induction of p21^Waf1^ relative to p53 activation. This disparity did not lead to differences in cell death, indicating that jnk2 expression does not mediate cellular response to DSBs but rather is specific to cell death in response to cell cycle initiation.

Given that the PyV MT/*jnk2*−/− cells showed less phosphorylation of the p53 Ser15 residue, we tested the role of ATM/ATR in replication induced cell death using caffeine (an ATM/ATR inhibitor) prior to and during FBS exposure. Figure 6B shows that caffeine inhibited FBS induced cell death in the PyV MT/*jnk2*−/− cells, whereas PyV MT/*jnk2*+/+ cells showed minimal apoptosis in any group. Caffeine's cytoprotection was associated with lower p21^Waf1^ expression and p53 Ser15 phosphorylation in the *jnk2* knockout cell line. Caffeine treatment also inhibited p53 phosphorylation in the PyV MT/*jnk2*+/+ cell line but p21^Waf1^ remained undetectable throughout (Figure 6C). These data support that cell cycle induced DNA damage associated with ATM or ATR activation leads to induction of p21^Waf1^ and cell death in the *jnk2* knockout cells.

We then focused our studies more closely on the DNA replication factor CDT1. CDT1 expression is necessary for replication fork progression during S phase. Geminin inhibits CDT1 to stall replication forks and allow G2/M transit. CDT1 degradation by proteases also facilitates this process. Lack of CDT1 inhibition/degradation or overexpression of CDT1 results in re-replication in some cell lines. In other cell lines, cell cycle check points inhibit re-replication by activating ATR/Chk1 responses. Collapsed replication forks or overt re-replication can lead to double strand breaks. ATM/p53 induction and increased p21Waf1 expression are responses that prevent or repair DNA damage [24].

**Figure 7:**
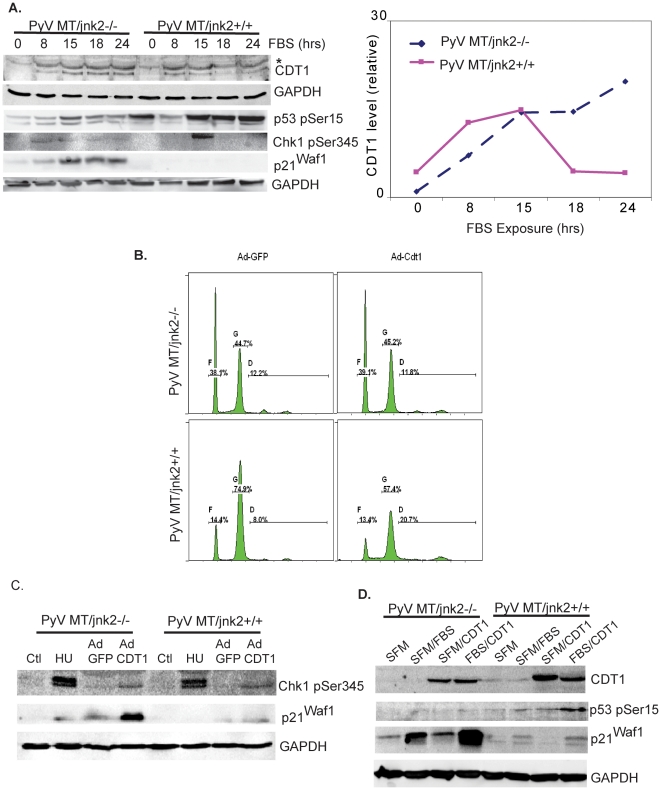
PyV MT/*jnk2*−/− cells experience replicative stress and increased p21^Waf1^ expression. **A**). Cells were serum starved and then harvested at different time points after 10% FBS stimulation to evaluate CDT1, p21^Waf1^, p-Chk1 (Ser345), and p-p53 (Ser 15) expression by western blot analysis using primary antibodies directed towards the indicated proteins. CDT1 expression at each time point was normalized to GAPDH and graphed for PyVMT/*jnk2+/+* and PyVMT/*jnk2−/−* cell lines; **B**). PyVMT/*jnk2+/+* and PyVMT/*jnk2−/−* cell lines were infected with either adenoviral-GFP or adenoviral-CDT1. Forty-eight hours later, cells were stained using PI with RNase, and then evaluated for cell cycle distribution using flow cytometry; **C**). Cells were infected with either adenoviral-GFP or adenoviral-CDT1 and harvested 24 hours later. Alternatively, cells were treated with hydroxyurea (HU 5 mM) for 24 hours and then harvested. Western blot analysis was used to measure pChk1 (Ser 345) and p21^Waf1^ expression. GAPDH was used to compare sample loading; **D**). Cells were infected with either adenoviral-GFP or adenoviral-CDT1 during 24 hours of serum starvation then stimulated with 10% FBS and harvested 24 hours later. pChk1 (Ser 345), p-p53 (Ser15), and p21^Waf1^ expression was evaluated using western blot analysis. GAPDH was used to compare sample loading.

As in previous studies, cells were serum starved and then stimulated with FBS. Endogenous Similarly, CDT1 expression was evaluated in a time dependent fashion along with p53 Ser15 phosphorylation and p21Waf1 expression. PyV MT/*jnk2*+/+ cells increased CDT1 expression after serum treatment which decreased after 18 and 24 hours, consistent with G2/M transit (Figure 7A). In contrast, PyV MT/*jnk2*−/− showed early and sustained induction of p21^Waf1^ and phosphorylated Chk1 which were concurrent with increased CDT1 expression which continued for at least 24 hours after FBS addition. These responses are indicative of replicative stress or prolonged S phase. In *jnk2* wildtype cells phosphorylation of p53 was preceded by Chk1 phosphorylation, CDT1 and p21^Waf1^ expression. Furthermore, Chk1 is only phosphorylated in PyV MT/jnk2+/+ cells in late S phase, consistent with normal S phase transit and the fact that Chk1 must become inactivated to recover from the checkpoint arrest [25], [26].

Overexpression of CDT1 initiates replication fork firing and induces a ATR/Chk1 response [27]. The effectiveness of CDT1 to induce replication fork firing is dependent upon its expression level and on its binding to the inhibitory protein geminin and/or degradation by Cul4-Ddb1^cdt2^ or SCF^SKP2^. Overexpression of CDT1 was used to more closely assess the role of JNK2 during replicative stress. Flow cytometric analysis showed that PyV MT/*jnk2*+/+ underwent re-replication when CDT1 was overexpressed compared to the PyV MT/*jnk2*−/− cells which did not (Figure 7B). To evaluate check point response during replicative stress, cells were left untreated, treated with hydroxyurea (HU, another agent inducing replicative stress by stalling replicative forks), or infected with adenoviral GFP or CDT1. Both cell lines responded to HU exposure and CDT1 over-expression by inducing phosphorylation of Chk1, an ATR substrate, showing that they both have an intact response to replicative stress. However, the PyV MT/*jnk2*−/− cells showed increased p21^Waf1^ in response to HU or CDT1 over-expression compared to the PyV MT/*jnk2*+/+ cells (Figure 7C). Cells were then serum starved and treated with FBS along with CDT1 overexpression which further induced p21^Waf1^ expression in the PyV MT/*jnk2*−/− cells with minimal effect on p53 Ser15 (Figure 7D). Conversely, PyV MT/*jnk2*+/+ cells showed a more blunted p21^Waf1^ response to FBS and/or CDT1 overexpression and additive p53 Ser15 phosphorylation when exposed to both. These data are all consistent with the interpretation that loss of *jnk2* expression is associated with replication stress check point activation through ATR/Chk1 and p21^Waf1^ in a p53 independent fashion.

**Figure 8:**
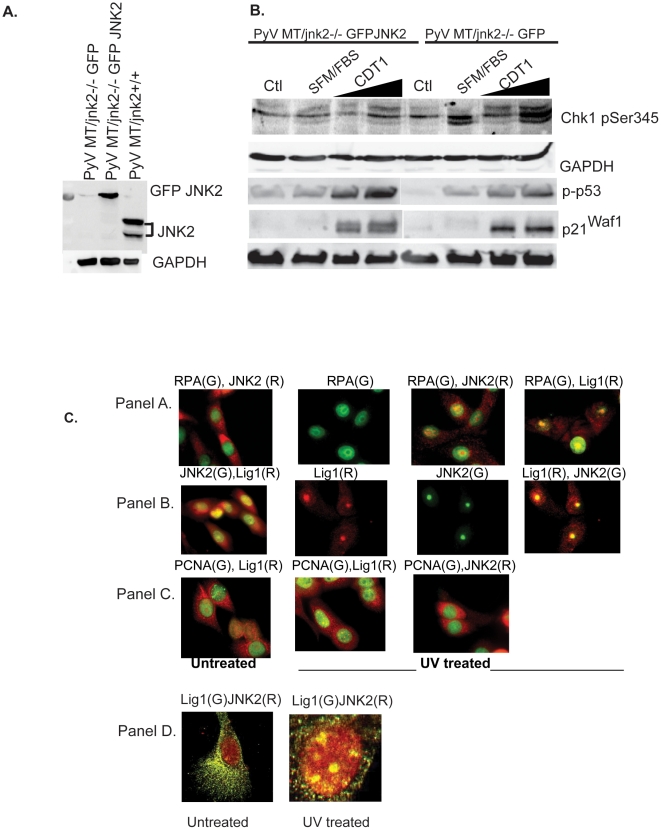
JNK2 is integral in sensing replicative stress and localizing at RPA coated lesions. **A**). PyVMT/*jnk2−/−* cells were infected with JNK2*α* retrovirus and selected using puromycin. GFP-JNK2 expression was measured using JNK2 primary antibody and PyVMT/*jnk2+/+* lysates as positive a control; **B**). PyVMT/*jnk2−/−* and PyVMT/*jnk2−/−GFP-JNK2a* expressing cells were infected with increasing doses of GFP-CDT1. Cells were processed as described in C). Cell lysates were analyzed for pChk1 (Ser 345), p53 (Ser 15) and p21^Waf1^. GAPDH was used to compare even sample loading. **C**). MCF10A cells were plated in chamber slides, untreated or treated with UV (10 J/m^2^), and fixed 2 hrs later. Cells were incubated with RPA, DNA Ligase 1 (Lig1), PCNA, or JNK2 primary antibodies, as indicated, followed by incubation with FITC or Texas Red secondary antibodies, (G) Green, (R) Red. Panel D includes images acquired using confocal microscopy. Co-localization was evaluated using color overlay.

To ascertain if the differences observed in these cell lines were due to *jnk2* expression, the PyV MT/*jnk2*−/− cells were transduced with GFP or GFP tagged JNK2α (the predominant JNK2 isoform) expressing retrovirus (Figure 8A). GFP and GFP JNK2α cells were then infected with increased inoculums of CDT1 adenovirus, and phosphorylated Chk1 expression was evaluated. Figure 8B shows that GFP expressing *jnk2* deficient cells showed an increase in Chk1 phosphorylation compared to control GFP expressing cells (consistent with ATR dependency of the response (Figure 6B)) which was associated with increased expression of p21^Waf1^. Interestingly, when p21^Waf1^ is separated using a higher percentage gel, a mobility shift is apparent in the GFP-JNK2 re-expressing cells, consistent with a post-translational change in p21^Waf1^ when JNK2 is expressed. On the other hand, phosphorylation of p53 Ser15 was lower in the GFP expressing cells compared to the GFP-JNK2 re-expressing cells, mirroring our previous observation with the PyV MT/*jnk2*+/+ cells. In summary, these data further validate that loss of JNK2 causes an early cell cycle checkpoint via p21^Waf1^ and Chk1 phosphorylation. Replicative stress induces p21^Waf1^, which delays or prevents re-replication, subsequent DSBs, and p53 response and repair. Without the appropriate induction of p53 response and repair functions, cells are unable to resume the cell cycle and undergo cell death.

These data suggest that JNK2 responds early or directly to replicative stress to influence DNA damage response and repair. During replicative or UV induced stress, RPA (a heterotrimeric protein) localizes to the DNA lesions or stalled replication forks. Phosphorylated Rad17 then translocates to the RPA modified, DNA strands [28], see refs [29], [30] for review. Subsequently, Rad17 recruits the 9-1-1 complex which induces DNA ligase 1 activity for repair [31]. For this experiment UV treatment was used to induce discrete DNA lesions to visualize foci microscopically. Cellular response to UV causes ssDNA lesions and initiates RPA coating of ssDNA. By causing ssDNA, UV treatment also leads to replication fork arrest and induces ATR activity [32]. Significantly, ATR phosphorylates p21^Waf1^ on Ser114 which is important for cdt2 degradation in response to UV treatment [33].

We hypothesized that JNK2 would localize to DNA breaks during UV induced DNA damage. For these studies, we aimed to evaluate normal DNA damage response by treating noncancerous, human MCF10A cells with UV irradiation. After UV treatment, RPA concentrated in specific areas of the nucleus consistent with its ability to coat ssDNA. After UV treatment, JNK2 and DNA Ligase 1 (Lig1) translocated from the cytoplasm to the nuclear RPA coated lesions (Figure 8C, Panel A). Panel B shows that JNK2 and DNA Ligase 1 co-localize to the same nuclear regions. The co-localization of JNK2 and DNA ligase 1 are specific to UV induced, RPA coated ssDNA lesions since they do not co-localize with PCNA (Panel C). Finally, confocal microscopy was used to further comfirm co-localization of JNK2 and DNA ligase 1 in response to UV treatment (Panel D). Together, these data support a role for JNK2 in sensing replicative stress and engaging subsequent repair mechanisms through p53 and other DNA repair responses.

## Discussion

Herein, we describe an oncogene induced mouse mammary tumor model where mice lacking *jnk2* experience higher tumor multiplicity and genomic instability. Unexpectedly, PyV MT/*jnk2−/−* had lower cell proliferation rates but there are likely several considerations to the finding. For example, the PyV MT/jnk2−/− tumors expressed less phosphorylated c-Jun (shown in Fig 1E) which induces a variety of proliferation related genes like cyclin D and c-myc. As noted, PyV MT*/jnk2−/−* tumors also express less DNA ligase 1. Its high expression level has been associated with increased proliferation rate in cancer cells [34], [35]. Lastly, increased p21^Waf1^ expression may contribute to less proliferation. Since cells cannot be synchronized *in vivo* due to constitutive expression of PyV MT, it is difficult to evaluate cell cycle changes in tumors. Therefore, we studied cell lines derived from these tumors. *In vitro* experiments mechanistically support that PyV MT/*jnk2−/−* cells experience replicative stress when stimulated to re-initiate the cell cycle. Cells lacking *jnk2* induce p21^Waf1^ and pChk1 prior to p53 activation to prevent re-replication. This response induces cell death in an ATR/ATM dependent fashion, as it is inhibited by caffeine.

Our *in vivo* data show that loss of *jnk2* leads to earlier and more frequent tumorigenesis. Tumors lacking *jnk2* showed more genomic instability, aneuploidy, and impaired DNA damage response/repair, perhaps due to a reduction or loss of DNA ligase 1 mediated response/repair during replicative stress. DNA ligase 1 expression increases during proliferation, and it binds to PCNA to join Okazaki fragments. DNA ligase 1 also mediates long-patch base excision repair and participates in the 9-1-1 (a checkpoint complex containing Rad9, Hus1 and Rad1) DNA damage response to repair single stranded DNA damage during replicative stress [36], [37], [38]. DNA Ligase 1 binds to Rad17 during S phase, concomitant with DNA damage [37]. Together, these properties suggest a dependence on DNA ligase 1 in tumor cells during oncogene induced replicative stress. Other investigators have also shown a link between JNKs and DNA ligase 1. Using siRNA targeting of JNK1 and JNK2, and a JNK pharmacologic inhibitor, *lig1* expression was changed, along with several other DNA repair genes, in reponse to cisplatin treatment [39]. In breast tumors and MEFs containing Rb/E2F mutations, DNA ligase 1 and other replication factors' expression are altered [40]. Together, these studies support that JNK2 is important in DNA damage response possibly via regulation of *lig1* expression, activation of ATR/Chk1/p21^Waf1^ response and co-localization with these proteins to ssDNA lesions.

Reduction in DNA ligase 1 function may not be the only event that contributes to the more tumorigenic phenotype observed. A reduction in a SWI/SNF related gene was also observed along with a reduction in a gene similar to *arp2*. Together, these proteins are known to be important for chromatin remodeling during replication and DNA damage [41], [42]. Futhermore, APC5 (product of *anapc5*) is part of a multisubunit ubiquitin ligase that mediates protein degradation during transit through G1 and mitosis. In Drosophila, IDA/APC5 mutants display aneuploidy without cell cycle arrest [43].

We speculate that the genomic instability observed is limited to the tumors rather than a general effect. *Jnk2−/−* mice (lacking expression of the PyV MT transgene) do not show overt phenotypes that would suggest the presence of genomic instability such as the *atm*−/− mice. To further evaluate the possibility that loss of *jnk2* may be causative to genomic instability, we also compared the ploidy status of Mouse Embryo Fibroblasts (MEFs) after inducing G_1_ arrest to evaluate DNA content. Both the *jnk2+/+* and *jnk2−/−* MEF lines were aneuploid (data not shown). This observation is likely due to the genomic instability of mouse cell lines with *in vitro* culture.

Previously, we reported that inhibition of JNK leads to endoreduplication in a p53 independent fashion using human breast cancer cell lines [9]. Similarly, MacCorkle and Tan specifically showed that JNK2 inhibition leads to polyploidy in human cancer cell lines [44]. We hypothesize that PyV MT and CDT1 overexpression cause replication under nonpermisive conditions to induce tumorigenesis. The absence of JNK2 further enhances replicative stress, genomic instability and tumorigenesis to ensue. Using established early-passage, cell lines derived from PyV MT tumors we were able to synchronize cells in G_1_ phase and follow cell cycle progression after FBS treatment (due to silencing of PyVMT expression). These studies showed that *jnk2* knockout cells induce p21^Waf1^ prior to p53 activation as shown by Ser15 phosphorylation. Overexpression of CDT1 further supported that the *jnk2* knockout cells underwent Chk1 and p21^Waf1^ induction in response to replicative stress, while re-expression of JNK2 inhibited Chk1 phosphorylation. During oncogene induced replicative stress and/or senescence, activation of p53 and Chk1 are increased to mediate damage repair, and p53 induced expression of p21^Waf1^ contributes to senescence. Surprisingly, PyV MT*/jnk2−/−* underwent cell death in response to replicative stress. This response may be due to an inefficient DNA damage response or repair associated with inadequate p53 or 9-1-1 reponse during replicative stress. Alternatively, others have reported that *c-jun* deficient cells undergo premature senescence resulting from DNA damage accumulation and inefficient repair [4]. Any or a combination of these responses would likely deter DNA repair and subsequently result in cell death. The presence of replicative stress and genomic instability is consistent with our *in vivo* model and reduced pH2AX and 53BP1 foci suggest a lack of DNA repair or response mechanisms in these tumors. We did not observe differences in cell death in tumors perhaps because cells grew asynchronously.

Consistently, *jnk2* knockout cells showed robust induction of p21^Waf1^ *in vitro* which did not correlate with p53 Ser15 phosphorylation. The ability of caffeine to inhibit cell death and p21^Waf1^ expression in the PyV MT*/jnk2−/−* cells supports a role for ATR in this response and places JNK2 as an intermediary between these kinases and p53/p21^Waf1^ effects. While p53 is usually attributed to an increase in p21^Waf1^ expression, other p53 independent mediators exist, including c-myc, Notch, ETS transcription factors, histone acetylation inhibitors, ATM, and c-Jun, amongst others [45]. Alternatively, our data mostly support that post-translational mechanisms contribute to this reponse. In JNK2 re-expressing cells, p21^Waf1^ underwent a mobility shift which may be due to phosphorylation. In fact, other investigators have reported p53 independent increases in p21^Waf1^ using similar models [46], [47]. There are a variety of explanations for the discordance between p53 and p21^Waf1^ responses during replicative stress. Most of these are related to mechanisms of p21^Waf1^ protein stability. For instance, MCM, geminin and CDT2 depletion lead to p21^Waf1^ accumulation and cell cycle checkpoint in a p53-independent manner [48], [49]. Abbas et al concluded that CDT2 facilitates DNA repair by degrading p21^Waf1^ [48]. Several kinases have been reported to phosphorylate p21^Waf1^, including Akt1 on Thr145 and Ser146 [50], [51] which inhibits p21^Waf1^ binding to PCNA and CDK2/4, indirectly enhancing cell proliferation. CDK2-cyclin E and GSK3 can also phosphorylate p21^Waf1^ on Ser130 and Thr57, respectively. Lastly, ATR phosphorylates p21^Waf1^ on Ser114 which is important for CDT2 degradation in response to UV treatment [33]. Phosphorylation of p21^Waf1^ reduces its stability. SCF^SKP2^ degrades phosphorylated p21^Waf1^ bound to CDK2 during G1/S and S phase transit. CRL4^cdt2^ also degrades phosphorylated p21^ Waf1^ when it's bound to PCNA during S phase or in response to UV treatment [52]. Our studies suggest that JNK2 may directly phosphorylate p21^Waf1^ or enhance activity of other kinases which phosphorylates p21^ Waf1^ to facilitate cell cycle transit.

Future studies will be aimed at understanding the influence of JNK2 in these responses and specifically addressing whether or not inhibition of JNK2 may be targeted therapeutically to enhance tumor cell death or senescence. Our data with JNK2 align with the paradoxial effects of oncogene expression wherein oncogene expression often faciliates cell replication but under certain conditions it ultimately induces a response that is incompatible with cell cycle transit.

## Materials and Methods

### Mouse tumorigenesis studies

FVB PyV MT mice were obtained from Dr. Bill Muller (McGill University, Montreal, Canada). All animal experiments were conducted according to institutional guidelines at the University of Colorado Health Sciences Center and the University of Texas, Austin. *Jnk2−/−* C57/BL6 mice and PyV MT mice were backcrossed into the Balb/C strain for over 10 generations. Female Balb/C mice with the genotypes PyV MT*/jnk2+/+*, PyV MT/*jnk2+/−*, and PyV MT/*jnk2−/−* were palpated three times weekly until the largest of palpable tumors (the “target” tumor) reached 150 mm^3^. At this point the mouse was euthanized, and all tumors, mammary glands, and lungs were harvested according to an approved IACUC protocol.

### Western blot analysis

Flash frozen tumors were homogenized in cold EB buffer (20 mM Tris-HCl, 250 mM NaCl, 3 mM EDTA, 0.05% Ipegal, 1 mM dithiothreitol, 0.368 mg/ml Na orthovanadate, 5 µg/ml leupeptin, 1 mM phenylmethylsulfonyl fluoride, and 17 µg/ml aprotinin) followed by centrifugation at 13,000 g to remove cellular debris. Fifty to 60 mcg of total cell lysate were resolved by SDS-PAGE and transferred to nitrocellulose. Western blot analyses were performed using primary antibodies to p53 overnight at 4°C, and later incubated with secondary antibody. Protein expression was detected using chemiluminescence with a Storm 860 Phosphorimager (GE Electronics). GAPDH expression was used as loading control for comparison of equal protein loading amongst samples.

### Primary tumor cell isolation and metaphase spreads

Tumor tissue was minced into 1 mm^3^ pieces with a sterile scalpel. Tissue fragments were washed with Dulbecco's Phosphate-Buffered Saline, and then re-suspended with 0.5 mg/ml collagenase A (Roche) containing serum-free media. Cells were incubated in a water bath shaker at 37°C, at 80 rpm overnight. The following day the suspension was centrifuged at 300 g for 5 min at 4°C. Cells were re-suspended in primary culture media (DMEM/F-12 (Mediatech Inc.) supplemented with 2% FBS (Benchmark), 1 mg/ml BSA (Sigma), 10 ug/ml insulin (Lilly) and 5 ng/ml EGF (Peprotech)). The cells were then cultured for 2 to 3 days at 37°C in a 5% CO_2_ incubator. Cells were filtered through a 70 micron Nylon mesh before splitting the second time. Cells from each tumor were used for cell cycle analysis and metaphase analysis.

### Metaphase analysis

Logarithmically growing primary tumor cells (passage 2–3) in 10 cm^2^ culture dishes were treated with colcemid (Sigma) for 55 minutes at 37°C in 5% CO_2_. Briefly, cells were collected using trypsin and re-suspended in 0.075 M KCl and incubated at room temperature for 20 minutes. The cells were re-suspended in ice cold Modified Carnoy's repeatedly four times and finally incubated overnight at −20°C. Cell suspensions were dropped onto glass slides and aged overnight at room temperature. Slides were then stained with Geimsa and trypsin + EDTA (0.25%). Metaphase spreads were visualized using a Nikon Diaphot 300 Microscope and a 100× oil objective.

### Cell cycle studies

Cell cycle distribution of primary tumor cells was measured using a Cytomics FC500 flow cytometer (Beckman Coulter) equipped with an argon laser with emission wavelength at 488 nm. Fluorescence of propidium iodide (PI) was collected using a 585/42 band-pass filter. A maximum of 50,000 events was collected from each sample. Analysis of the cell cycle compartments was carried out using CXP analysis software.

### Histology and Immunohistochemistry

For tumor sections, 5 micron, paraffin-embedded tissue sections were either stained in hematoxylin and eosin or used for immunohistochemistry with Ki-67 (Neomarkers), cleaved caspase-3, pH2AX (S139) (Calbiochem), p-cJun (S63) (Cell Signaling), or 53BP1 (Bethyl Laboratories) as indicated. Expression of proteins was detected by using DAB (Vector Labs) or fluorescence. Hematoxylin and propidium iodide staining were used as nuclear markers. Fluorescent images were captured on a Nikon Diaphot inverted microscope using a CoolSnapfx camera and ImagePro 6.1 software for color overlay.

### qPCR of lig1, and anapc5

*Lig1* and *anapc5* measurements were generated from target tumors. 18S was used as a loading control for tumor samples. Samples were amplified using SYBR green fluorescence on a Stratagene Mx3005p (Agilent Technologies Company).

### Array Comparative Genomic Hybridization (aCGH)

Genomic DNA was isolated from tumors using Gentra Puregene Tissue Kit (Qiagen). Samples were then hybridized and data generated using Roche Nimblegen services and technology.

### Cell lines and JNK2 Overexpression

PyV MT*/jnk2+/+* and PyV MT/*jnk2−/−* cell lines were derived from mammary tumors. Cells were maintained in DMEM/F12 (1∶1) medium (Mediatech Inc.) supplemented with 10% FBS (Benchmark), 10 ug/ml insulin (Lilly), 5 ng/ml EGF (Peprotech), 5 µg /ml linoleic acid (Biosource), 50 µg/ml gentamicin, 100 units/ml penicillin, and 100 µg/ml streptomycin (Invotrogen). PyV MT*/jnk2−/−* cells were reconstituted with GFP-tagged mouse JNK2α protein, or GFP empty vector. The cells were cultured in medium, containing 2 µg/ml puromycin. Cells were synchronized in defined serum free medium (DMEM/F12 medium containing 2 µg/ml transferrin, 2 µg/ml human fibronectin (BD Biosciences) and 1× trace elements (Biosource)) for 24 hours.

### Antibodies and Western Blot Analysis

Anti-53BP1 (Bethyl laboratory Inc., Montgomery, TX), anti-JNK2 (D2) and anti-CDT1 (Santa Cruz Biotechnology, CA), anti-cleaved caspase 3 (Cell Signaling Technology, MA), anti-p53 (Imgenex, San diego, CA), anti-Rb, anti-p21^Cip1^ (BD Pharmingen, San Jose, CA), and mouse anti-GAPDH (Advanced ImmunoChemical Inc.) antibodies were used for western blot analysis. Anti phospho-c-Jun (Ser63), anti-phospho-p53 (Ser15), anti- phospho-CHK1 (Ser345), anti- phospho-H2AX (Ser139) antibodies were used for detecting the specific phosphorylated proteins. Proteins were detected by enhanced chemiluminescence (ECL) kit (Amersham Pharmacia Biotech, NJ).
